# Trends in Receipt of Help at Home After Hospital Discharge Among Older Adults in the US

**DOI:** 10.1001/jamanetworkopen.2021.35346

**Published:** 2021-11-30

**Authors:** Eric Bressman, Norma B. Coe, Xinwei Chen, R. Tamara Konetzka, Rachel M. Werner

**Affiliations:** 1 Leonard Davis Institute, University of Pennsylvania, Philadelphia; 2 Department of Medicine, Perelman School of Medicine, University of Pennsylvania, Philadelphia; 3 Corporal Michael J. Crescenz VA Medical Center, Philadelphia, Pennsylvania; 4 Department of Medical Ethics and Health Policy, Perelman School of Medicine, University of Pennsylvania, Philadelphia; 5 Department of Public Health Sciences, University of Chicago, Chicago, Illinois; 6 Department of Medicine, University of Chicago, Chicago, Illinois

## Abstract

**Importance:**

With declining use of institutional postacute care, more patients are going directly home after hospital discharge. The consequences on the amount of help needed at home after discharge are unknown.

**Objective:**

To estimate trends in the frequency and duration of receipt of help with activities of daily living (ADLs) among older adults discharged home.

**Design, Setting, and Participants:**

Repeated cross-sectional study of a national sample of community-dwelling older adults who returned home after hospital discharge from 2011 to 2017. Participants included respondents to National Health and Aging Trends Study (NHATS), an annual population-based, nationally representative survey of Medicare beneficiaries, who were 69 years or older and were discharged from an acute care hospital to home during the years of the study. A nationally representative sample was estimated using NHATS’ analytic weights. Unweighted frequencies and weighted and unweighted percentages are reported. The analysis was conducted from September 2020 to October 2021.

**Exposures:**

Discharge from an acute care hospitalization.

**Main Outcomes and Measures:**

Receipt of help with ADLs during the 3 months after hospital discharge.

**Results:**

Of the 3591 survey participants who were discharged home from an acute care hospital during the study period, 53.3% were female, 54.8% were married or living with a partner, and the mean (SD) age was 78.5 (7.0) years. Of these, 1710 (44.1%) reported receiving help within 3 months of discharge. Compared with people not receiving help, those receiving help were older (mean [SD] years, 79.7 [7.5] years vs 77.6 [6.3] years), had worse self-rated health at baseline (47.1% with fair or poor health vs 26.5%) and were more likely to have dementia (21.8% vs 5.5%). The percentage of respondents who reported receiving help increased during the study period from 38.1% of hospital discharges in 2011 to 51.5% in 2017. For those who were independent in their ADLs before hospitalization, the percentage receiving help after discharge more than doubled over the study period increasing from 9.3% receiving help in 2011 to 31.8% in 2017. Among patients who did not receive Medicare-reimbursed home health, the percentage receiving help also increased from 22.1% to 28.1%. Among those who received help after discharge, the need for help slowly declined to prehospitalization levels over the ensuing 9 months.

**Conclusions and Relevance:**

In this cross-sectional study, older adults’ receipt of help at home after hospital discharge increased from 2011 to 2017, including patients relying on non-Medicare funded sources of care. As payers steer patients away from inpatient postacute care facilities, policymakers will need to pay attention to this shifting burden of care.

## Key Points

**Question:**

How has the need for help at home after hospital discharge among older adults changed in the context of declining use of institutional postacute care?

**Findings:**

In this cross-sectional study of 3591 participants, the prevalence of older adults receiving help at home during the posthospital period increased from 2011 to 2017. This included increases in non-Medicare reimbursed sources of help.

**Meaning:**

As the use of institutional postacute care declines over time, additional attention will need to focus on whether the shift home increases the burden of care on family and friends and whether additional support is needed to support home-based care.

## Teaser

This cross-sectional study estimates trends in the frequency and duration of receipt of help with activities of daily living among older adults discharged home.

## Introduction

Hospitalization for acute illness often results in functional declines for older adults, requiring a period of rehabilitation and recovery after hospital discharge. For decades, this postacute recovery was frequently provided in inpatient settings,^1^ most commonly in skilled nursing facilities and inpatient rehabilitation facilities. However, given the high costs of institutional postacute care,^2^ reducing its use has recently been a common goal across payers, including Medicare and commercial payers. With the implementation of alternative payment models, such as episode-based payment and accountable care organizations, there has been a decline in institutional postacute care use, shifting more posthospital care home.^3^

Recovering from a hospitalization at home is often aligned with patients’ preferences^4^ and lowers health care spending.^5^ However, older adults often have functional limitations after hospitalization, requiring ongoing (although short-term) help with activities of daily living, such as feeding and bathing themselves and getting out of bed and moving around their homes. Home health care workers may provide some of this assistance, and the use of home health care after hospital discharge is common.^6^ However, home health visits alone are insufficient to provide the frequent care that some older adults need. In these cases, care is often provided by unpaid caregivers,^7,8^ usually family members or friends, at significant costs to the caregiver and the family.^9^

With the recent declines in institutional postacute care, it is unknown but important to understand how in-home care has changed for older adults after hospital discharge, particularly as we seek to understand the full costs and benefits of shifting care home after hospital discharge. In this article, we estimate trends in the frequency and duration of receipt of help with activities of daily living (ADLs) among patients discharged home from 2011 to 2017.

## Methods

This cross-sectional study was reviewed and approved by the institutional review board at the University of Pennsylvania and was granted a waiver from informed consent because of the use of existing data. This study followed the Strengthening the Reporting of Observational Studies in Epidemiology (STROBE) reporting guidelines.

### Data and Study Cohort

This study used data from the National Health and Aging Trends Study (NHATS) linked to Medicare claims and assessment data. The NHATS is a population-based, nationally representative survey of Medicare beneficiaries ages 65 years and older. It is the only survey that captures individuals’ receipt of assistance with activities relating to mobility (ie, getting around inside and outside the home and getting out of bed) and self-care (ie, eating, getting cleaned up, toileting, or getting dressed) on a monthly basis, which enables us to more precisely measure the receipt of help with these activities in relation to hospital discharge.

More specifically, NHATS interviews are conducted annually with study participants or their designated proxies if the participant is unable to respond. For the activities of mobility and self-care, respondents are asked whether they received help with these activities since the last survey and, if so, the exact months in which they received help. By linking together rounds of the survey, NHATS allows a complete representation of monthly receipt of help with mobility and self-care activities across survey rounds for most sample persons.^10^ For other activities (eg, household activities and medical care), respondents are only asked if they received help in the month before the survey.

NHATS data for participants enrolled in fee-for-service Medicare are linked to Medicare claims, assessment, and enrollment data, allowing us to identify all acute care hospitalizations for NHATS participants, including hospital discharge dates. Using 7 rounds of NHATS data, collected annually from 2011 to 2017, we included all NHATS respondents who were Medicare fee-for-service participants with an acute care hospital discharge. Because we focus our analysis on the receipt of help in home-based settings, we excluded all participants who were discharged to a skilled nursing facility, inpatient rehabilitation facility, or another institutional setting within 3 days of hospital discharge. To mitigate the confounding effects of an aging study population over our study period, we included respondents who were 69 years or older in the survey year, rather than the 65 years or older inclusion criteria used by NHATS, which would have resulted in the minimum age in our sample increasing in each year of the study until the study sample was replenished. We chose 69 years as the lower limit for age because the NHATS sample was replenished after 4 years of survey rounds (in 2015), making the minimum age 69 years across all years. We then included all community-dwelling survey respondents who had valid non–missing responses to at least 1 question pertaining to receipt of help with either mobility or self-care. eTable 1 in the Supplement contains details on missing data and study sample size at each exclusion step; less than 4% of respondents were missing data. For participants who had more than 1 hospitalization between survey rounds, we randomly selected 1 hospital discharge per survey round.

We further defined 3 subgroups based on patient factors. First, we excluded respondents who reported receiving Medicare-reimbursed home health visits after hospital discharge to test whether trends in receiving help after hospital discharge were driven by increased home health use or another source, such as informal caregiving. NHATS does not ask respondents who provided help with mobility or self-care, and trends in receiving this help may be partially due to help from home health aides, who may address ADL-activities. Therefore, we excluded those with 1 or more visits from a home health agency during the 90 days after hospital discharge using NHATS-linked home health visit data from Medicare’s Outcome and Assessment Information Set data. Because some Medicare–reimbursed home health visits primarily cover skilled needs (eg, skilled nursing, physical, occupational, and speech language therapy) and not ADL-related activities, the number of respondents who reported receiving help but did not have home health visits and the number of respondents with home health visits do not sum to the number of respondents who reported receiving any help in NHATS (eTable 2 in the Supplement).

Second, we examined the subset of participants who were independent in their ADLs before hospitalization, defined as those who did not report receiving help with mobility or self-care in the month prior to hospitalization. We did this to define a cohort for whom postdischarge help was new and temporally associated with a hospitalization. Third, to investigate the role of cognitive impairment in trends in receiving help, we examined the subset of participants who were classified as having probable dementia in the survey before hospitalization.^11^

### Measures

We report baseline characteristics of our study cohort, including sociodemographic characteristics, health characteristics, and information related to their hospitalization. Sociodemographic characteristics included study participants’ age at the time of hospitalization, sex, race and ethnicity, and marital status. Health characteristics included self-rated health, cognitive status (based on an NHATS-derived algorithm for classification by dementia status^11,12^), and independence in 4 functional domains (ie, mobility, self-care, household activities, and medical care), all measured at the most recent survey interview prior to hospitalization. Hospitalization information included length of hospital stays and the prevalence of the 5 most common diagnosis-related groups in our study sample.

Race and ethnicity were self-reported and included in this study as part of the sociodemographic description of the cohort. Race data were collected in the following categories: Alaska Native, American Indian, Black or African American, Native Hawaiian, Pacific Islander, White, and other. Ethnicity data were collected in the categories of Cuban American, Mexican American or Chicano, Puerto Rican, and other. Race and ethnicity data were combined into categories and included in the survey data as Hispanic, non-Hispanic Black, non-Hispanic White, non-Hispanic other, more than 1, and refused or do not know. The category non-Hispanic other included individuals who identified as Alaskan Native, American Indian, Asian, Native Hawaiian, Pacific Islander, or other.

Our main outcome of interest was whether each participant reported receiving help in mobility or self-care activities during the 3 months after hospital discharge. We summarized help with mobility or self-care as a composite measure of help with ADLs. ADLs include walking, transferring, feeding, dressing, toileting, and bathing, and are well encapsulated by these 2 domains.

We also examined a secondary outcome of the duration of receiving help after hospital discharge. To do so, we measured the receipt of help with ADLs in the month before hospitalization (as a baseline) and in each month after discharge for 9 months.

### Data Analysis and Estimation

We described our sample of survey respondents in all survey rounds, describing the total population and stratifying by those who did and did not report receiving help after hospital discharge. We also described survey respondents by survey round in eTable 3 in the Supplement. The analysis was conducted from September 2020 to October 2021.

We estimated the annual rate of receiving help with ADLs in the 3 months after hospital discharge for repeated cross-sections from each survey round among all discharges and for our 3 subgroups: those who did not receive home health; those who were independent in their ADLs prior to hospitalization; and those with probable dementia (compared with no or possible dementia). Finally, among respondents who reported receiving help after discharge, we estimated the rate of receipt of help by month, beginning with the month before hospitalization until 9 months after discharge among the same groups defined earlier: all discharges, those who did not receive help prior to hospitalization, and those with probable dementia. Within these groups, we also estimated the monthly receipt of help with ADLs among those who did not have Medicare-reimbursed home health after hospital discharge.

Because our goal was to describe observed trends, we did not formally test whether trends were statistically different from zero. In our main results, we estimate a nationally representative sample using NHATS’ analytic weights, which adjust for differential probabilities of selection and nonresponse to make national estimates. We present unweighted frequencies and weighted percentages in the results. To test whether our results were sensitive to using weights, we recalculated all percentages without weights.

To test whether our results could be explained by sample selection bias caused by differential attrition of people who did not receive help at home between annual surveys we did 2 things. First, because the NHATS sample was replenished in 2015 with new participants, we examined whether the replenishment sample had lower rates of receipt of help at home after hospital discharge than the original sample in the years 2015 to 2017. Second, in cohorts where the replenishment sample did have lower rates of receiving help at home after hospital discharge, we directly examined whether those who received help had lower rates of attrition than those who did not receive help with ADLs at home after hospital discharge.

## Results

We included 3591 hospital discharges among survey respondents, representing an estimated 17.9 million discharges nationally, of whom, 53.3% were female, the mean (SD) age was 78.5 (7.0) years, 8.3% were non-Hispanic Black individuals, 81.3% were non-Hispanic White individuals, 54.8% were married or living with a partner, and 12.8% had probable dementia. Across this cohort, 44.1% reported receiving help with ADLs during the posthospital discharge period (Table 1). Overall, just more than one-quarter of the cohort had 1 or more home health visits (Table 2). Medicare-reimbursed home health visits were more common among those who reported receiving help with ADLs compared with those who reported not receiving help (42.0% vs 17.5%). Furthermore, 58% of those who reported receiving help did not have any home health visits.

**Table 1.** Characteristics of Study Cohort, Including All Patients Discharged Home From an Acute Care Hospital

Characteristics	No. (weighted %)		
	Overall Cohort	Cohort receiving help	Cohort not receiving help
Observations, No.	3591	1710	1881
Estimated population size	17 903 595	7 891 029	10 012 566
Female	2015 (53.3)	1092 (60.1)	923 (48.0)
Male	1576 (46.7)	618 (39.9)	958 (52.0)
Age, mean (SD), y	78.5 (7.0)	79.7 (7.5)	77.6 (6.3)
Race and ethnicity			
Hispanic	165 (5.4)	97 (6.4)	68 (4.5)
Non-Hispanic Black	797 (8.3)	409 (9.0)	388 (7.7)
Non-Hispanic White	2475 (81.3)	1114 (78.2)	1361 (83.8)
Other^a^	154 (5.0)	90 (6.4)	64 (3.9)
Married or living with partner	1647 (54.8)	722 (53.9)	925 (55.5)
Self-rated health			
Excellent	158 (6.7)	55 (4.6)	103 (8.3)
Very good	525 (22.6)	188 (16.9)	337 (27.4)
Good	911 (34.4)	411 (30.7)	500 (37.4)
Fair	747 (25.1)	425 (30.8)	322 (20.3)
Poor	312 (10.8)	216 (16.3)	96 (6.2)
Independent in			
Mobility	1877 (73.2)	738 (59.0)	1139 (84.8)
Self-care	2143 (82.7)	888 (70.6)	1255 (92.6)
Household activities	1531 (62.8)	485 (42.4)	1046 (79.4)
Medical care	2101 (81.9)	850 (69.5)	1251 (92.1)
Cognitive status			
No or possible impairment	2179 (86.4)	923 (77.2)	1256 (93.9)
Probable dementia	476 (12.8)	371 (21.8)	105 (5.5)
Hospital length of stay in days, mean (SD)	3.5 (2.9)	3.9 (3.2)	3.2 (2.7)
Diagnosis related group			
Major joint replacement (n = 470)	162 (6.6)	88 (8.6)	74 (5.0)
Septicemia or severe sepsis (n = 871)	102 (2.6)	68 (3.5)	34 (1.9)
Esophagitis, gastroenteritis and miscellaneous digestive disorders (n = 392)	93 (2.4)	54 (2.8)	39 (2.0)
Simple pneumonia & pleurisy (n = 194)	75 (2.1)	29 (1.7)	46 (2.4)
Cellulitis (n = 603)	69 (2.1)	22 (1.4)	47 (2.6)

**Table 2.** Summary of Posthospitalization Care in the Study Cohort, Including All Patients Discharged Home From an Acute Care Hospital

Characteristics of Posthospital Care	No. (weighted %)		
	Overall Cohort	Cohort receiving help	Cohort not receiving help
Observations, No.	3591	1710	1881
Estimated population size	17 903 595	7 891 029	10 012 566
Received any help within 3 mo after discharge	1710 (44.1)	1710 (100.0)	0
Had home health visits within 3 mo after discharge	1116 (28.3)	744 (42.0)	372 (17.5)
Received help but did not have home health visits within 3 mo after discharge	966 (25.6)	966 (58.0)	0

Compared with patients not receiving help with ADLs, those who reported receiving help with ADLs were more likely to be female (60.1% receiving help vs 48.0% not receiving help) and older (mean [SD] age, 79.7 [7.5] years receiving help vs 77.6 [6.3] years not receiving help) and less likely to be non-Hispanic White (Table 1). Prior to hospitalization, patients receiving help with ADLs also had worse self-rated health, lower functional independence, and were more likely to be classified as having probable dementia. Their hospital stays tended to be slightly longer and were more likely to have been related to a major joint replacement or sepsis. The study cohort characteristics were generally consistent across survey rounds although the proportion of non-Hispanic Black survey respondents decreased over time, fewer people reported being in fair or poor health, and the proportion of individuals hospitalized for a major joint replacement increased over time (eTable 3 in the Supplement). Unweighted sample characteristics showed a slightly older cohort and a higher percentage of non-Hispanic Black individuals, with similar differences between those who did and did not receive help (eTable 4 in the Supplement).

### Trends in Receipt of Help After Hospital Discharge

**Figure 1.**
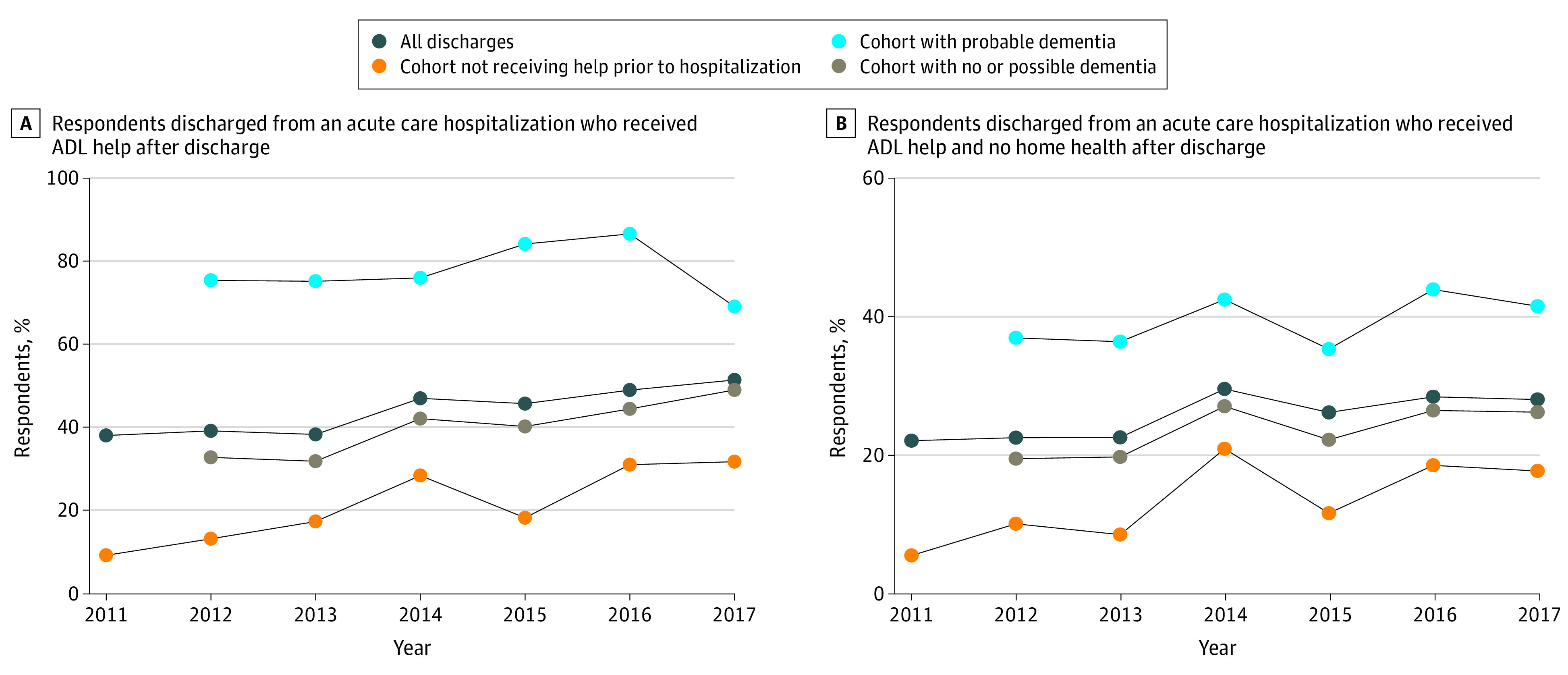
Percentage of People Discharged From an Acute Care Hospitalization Who Reported Receiving Help With Activities of Daily Living (ADLs) Cognitive status was measured in the survey prior to hospitalization and is thus missing in the first year of the study period. All percentages were weighted.

The rate of receiving help with ADLs during the posthospital period increased over the study period from 38.1% of those discharged home in 2011 to 51.5% in 2017, a relative increase of 35% (Figure 1A). Among those who were independent in their ADLs prior to hospitalization, the rate of receiving help with ADLs after hospital discharge more than tripled over the study period—from 9.3% to 31.8%, a relative increase of 242.9%.

Among patients who did not receive Medicare-reimbursed home health these trends were similar (Figure 1B). The rate of receipt of ADL help increased from 22.1% in 2011 to 28.1% in 2017, a relative increase of 26.9%. For those who were independent in their ADLs prior to hospitalization, this rate increased from 5.5% to 17.7%, a relative increase of 220.2%.

Among patients with probable dementia, the increase in the rate of receipt of help with ADLs was smaller in both the cohort of all discharges and those not receiving Medicare-reimbursed home health (Figure 1). For those with no or possible dementia, the rate of receipt of help with ADLs increased more. In the cohort of all discharges, the rate of help with ADLs increased from 32.8% to 49.1%, a relative increase of 49.4% (Figure 1A). In the cohort not receiving Medicare-reimbursed home health, the rate of help with ADLs increased from 19.5% to 26.2%, a relative increase of 34.4% Figure 1B).

These trends were similar in the unweighted sample (eFigure 1 in the Supplement). The 2015 replenishment sample had similar or higher rates of receipt of help with ADLs as the original 2011 sample in the 2015 to 2017 surveys for most cohorts, suggesting that observed upward trends were not due to sample attrition (eFigure 2 in the Supplement). The exception to this was the cohort who did not receive help before hospitalization, which had lower rates of receiving help after hospital discharge, particularly in 2015. In this cohort, the rate of attrition in the 2016 survey was higher among people who did receive help at home after hospital discharge compared with those who did not receive such help (4 of 12 [33%] vs 21 of 124 [17%]), suggesting that observed upward trend was not due to sample attrition.

### Duration of Receipt of Help After Hospital Discharge

**Figure 2.**
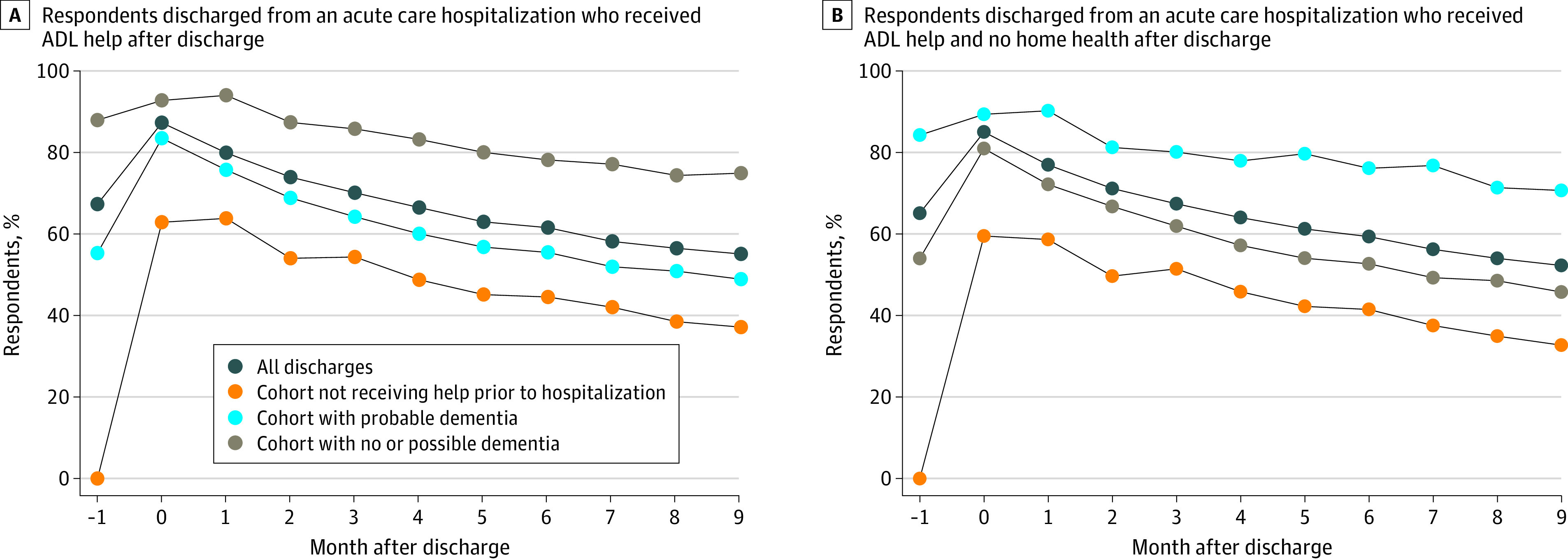
Percentage of Respondents Who Reported Receiving Help With Activities of Daily Living (ADLs) in the 9 Months After Hospital Discharge Month 0 is the month of hospital discharge. All percentages were weighted.

Limiting the cohort to those who reported receiving help with ADLs in the 3 months after hospital discharge, that rate of help was highest in the month of hospitalization in both the cohort of all discharges and the cohort not receiving Medicare-reimbursed home health (Figures 2A and 2B). The prevalence of receiving help with ADLs then slowly declined over the ensuing months in both groups and reached prehospitalization levels of ADL help after 3 months.

For those who had not been receiving help in the month before discharge, this trend was similar but the percentage of those needing help with ADLs remained greater than 30% in both groups by the ninth month, with 37.2% of people still receiving help with ADLs at 9 months after discharge in the cohort of all discharges and 32.7% in the cohort not receiving Medicare-reimbursed home health.

The trend was also similar among those with no or possible dementia. Rates of receiving help with ADLs remained high in every month for those with probable dementia. These findings were similar in the unweighted sample (eFigures 3A and 3B in the Supplement).

## Discussion

Given the high costs of institutional postacute care,^2,13^ recent health care payment policies have focused on reducing its use.^3^ With health care shifting away from Medicare-reimbursed institutional postacute care, it is essential to understand where and to whom this care is being shifted.

To our knowledge, this study provides the first description of trends in the receipt of help with ADLs at home among older adults discharged from the hospital. We found that the prevalence of those receiving help at home during the posthospital period increased from 2011 to 2017.

Over this same period, Medicare-reimbursed institutional postacute care has been declining.^6^ Our results of increasing help with ADLs in the posthospital period may reflect the shifting location of care as patients are steered away from inpatient postacute care facilities after hospital discharge. It also reflects a shifting of payment for postdischarge care—away from Medicare-reimbursed care for institutional postacute care and home health and toward alternative and nonreimbursed sources of home-based care. This substantial increase in non–Medicare-covered help at home is likely being provided by unpaid caregivers, consistent with the increase in unpaid caregiving during this period generally.^14^

Numerous studies have documented the decline in institutional postacute care spending with the implementation of bundled payments^3,15^ and accountable care organizations,^16^ which have translated into spending reductions for Medicare money. However, these prior calculations have failed to consider the societal costs of shifting posthospital discharge care out of institutions and into homes. Our study suggests these costs are being borne by families, either through privately paid or Medicaid-reimbursed help in the home or through informal (ie, unpaid) caregiving by families and friends. While we are unable to distinguish the exact source from which patients report receiving help, we do know it is not Medicare-reimbursed care. This suggests that while Medicare has reported financial cost savings from alternative payment models that more frequently send patients home after hospital discharge, these payment models may be increasing societal costs by shifting the burden of care from Medicare to other sources including caregivers.

It is particularly concerning that this care may be shifted to unpaid caregivers. Caregiving burden is a large and growing concern in the United States and is associated with physical, emotional, economic, and health-related stresses.^17,18,19,20,21^ In a recent US study, an estimated 34 million adults provided unpaid care to an older adult in the prior year,^7,8^ making caregiving an important challenge.

While unpaid caregivers may be providing valuable support to family and friends during this critical posthospital recovery period, it is not clear whether they are equipped to provide sufficient support alone. Caregivers often receive little or inadequate training and are poorly integrated into care teams. The impact of unpaid caregiving on subsequent cost of care and use of acute care services remains unknown.^22,23,24^ Of equal concern is the burden placed on the caregivers themselves. There is a growing body of evidence that this burden adversely impacts caregivers’ well-being,^17,20,25^ and it is disproportionately borne by women.

While we found an increasing rate of postdischarge receipt of help with ADLs in the home, whether it is directly related to payment reforms targeting the use of postacute care is unknown. Future analyses should be directed toward this very question, as it is integral to gaining a more complete picture of the effects of payment reform, including who is caring for older adults during this critical recovery period, and at what cost. The work at hand lays the foundation for a better understanding of these shifting burdens.

### Limitations

This study has several limitations. We relied on claims data for Medicare fee-for-service patients, and therefore lacked information on individuals who were enrolled in Medicare Advantage. Additionally, survey data may be subject to recall and nonresponse bias, although response rates in NHATS are generally high and the self-reported responses have been shown to be reliable.^26^ While we are able to report data on whether survey respondents received help by month, we do not have information on who is providing this help. We did not conduct any form of statistical testing. Additionally, we limited the sample to those not receiving Medicare-reimbursed home health care but cannot be certain whether the care is paid for out of pocket, by a non-Medicare insurer, or is unpaid.

## Conclusions

In this cross-sectional study, older adults’ receipt of help at home after hospital discharge increased substantially from 2011 to 2017. This included an increasing prevalence of help provided by individuals other than Medicare-covered home health care professionals. As payers steer patients away from inpatient postacute care facilities, attention will need to be paid to this shifting burden of care, ensuring a more complete accounting of health care spending under alternative payment models (including societal costs), and, if care is being shifted to unpaid caregivers, policies to support these individuals.

## Notes

Supplement. **eTable 1.** Number of Respondents Included After Each Exclusion Criteria by Round **eTable 2.** Summary of Posthospitalization Care in Study Cohort, Including All Sample Persons Discharged Home From an Acute Care Hospital (Unweighted Percentages) **eTable 3.** Characteristics of Study Cohort and Care Received After Hospital Discharge, Including All Patients Discharged Home From an Acute Care Hospital, by Year (Weighted Percentages) **eTable 4.** Characteristics of Study Cohort, Including All Patients Discharged Home From an Acute Care Hospital (Unweighted Percentages) **eFigure 1.** Unweighted Percentage of Sample Persons Discharged From an Acute Care Hospitalization Who Received ADL Help After Discharge and Persons Who Received ADL and No Home Health Care After Discharge, by Year **eFigure 2.** Weighted Percentage of Sample Persons Discharged From an Acute Care Hospitalization Who Received ADL Help After Discharge, by Year and by Survey Wave **eFigure 3.** Unweighted Percentage of Individuals Who Reported Receiving Help in Each Month After Hospital Discharge and Those Who Received Help and Did Not Have Any Medicare-Reimbursed Home Health Visits in Those 3 Months
