# Supplementary material for: Trends in Receipt of Help at Home After Hospital Discharge Among Older Adults in the US
Source: JAMA Netw Open. 2021 Nov 30;4(11):e2135346. doi: 10.1001/jamanetworkopen.2021.35346 (PMC8634055; doi:10.1001/jamanetworkopen.2021.35346)
Supplement: Supplement. — eTable 1. Number of Respondents Included After Each Exclusion Criteria by Round eTable 2. Summary of Posthospitalization Care in Study Cohort, Including All Sample Persons Discharged Home From an Acute Care Hospital (Unweighted Percentages) eTable 3. Characteristics of Study Cohort and Care Received After Hospital Discharge, Including All Patients Discharged Home From an Acute Care Hospital, by Year (Weighted Percentages) eTable 4. Characteristics of Study Cohort, Including All Patients Discharged Home From an Acute Care Hospital (Unweighted Percentages) eFigure 1. Unweighted Percentage of Sample Persons Discharged From an Acute Care Hospitalization Who Received ADL Help After Discharge and Persons Who Received ADL and No Home Health Care After Discharge, by Year eFigure 2. Weighted Percentage of Sample Persons Discharged From an Acute Care Hospitalization Who Received ADL Help After Discharge, by Year and by Survey Wave eFigure 3. Unweighted Percentage of Individuals Who Reported Receiving Help in Each Month After Hospital Discharge and Those Who Received Help and Did Not Have Any Medicare-Reimbursed Home Health Visits in Those 3 Months [file jamanetwopen-e2135346-s001.pdf]

Supplemental Online Content

Bressman E, Coe NB, Chen X, Konetzka RT, Werner RM. Trends in receipt of help at home after hospital discharge among older adults in the US. *JAMA Netw Open*. 2021;4(11):e2135346. doi:10.1001/jamanetworkopen.2021.35346

eTable 1. Number of Respondents Included After Each Exclusion Criteria by Round
eTable 2. Summary of Posthospitalization Care in Study Cohort, Including All Sample Persons Discharged Home From an Acute Care Hospital (Unweighted Percentages)
eTable 3. Characteristics of Study Cohort and Care Received After Hospital Discharge, Including All Patients Discharged Home From an Acute Care Hospital, by Year (Weighted Percentages)
eTable 4. Characteristics of Study Cohort, Including All Patients Discharged Home From an Acute Care Hospital (Unweighted Percentages)
eFigure 1. Unweighted Percentage of Sample Persons Discharged From an Acute Care Hospitalization Who Received ADL Help After Discharge and Persons Who Received ADL and No Home Health Care After Discharge, by Year
eFigure 2. Weighted Percentage of Sample Persons Discharged From an Acute Care Hospitalization Who Received ADL Help After Discharge, by Year and by Survey Wave
eFigure 3. Unweighted Percentage of Individuals Who Reported Receiving Help in Each Month After Hospital Discharge and Those Who Received Help and Did Not Have Any Medicare-Reimbursed Home Health Visits in Those 3 Months

This supplemental material has been provided by the authors to give readers additional information about their work.

© 2021 Bressman E et al. *JAMA Network Open.*

**eTable 1.** Number of Respondents Included After Each Exclusion Criteria by Round							
	**2011**	**2012**	**2013**	**2014**	**2015**	**2016**	**2017**
**START: All NHATS respondents**	8245	7075	5799	4737	8332	7274	6310
**1. After excluding if age <69**	7097	6441	5527	4713	7480	6787	6083
**2. After excluding if lived in nursing home or care facility**	6070	5527	4769	4073	6534	5937	5353
**3. After excluding if did not have any Medicare FFS claims**	5096	4656	3986	3360	5016	4518	4001
**4. After excluding if did not have an inpatient claim within 365 days prior to the survey, or prior to the date of death**	809	932	816	624	842	801	779
**5. After excluding if not hospitalized in acute care hospital or critical access hospital or was not discharged alive**	802	897	783	602	823	774	749
**6. After excluding if went to an institutional setting after hospital discharge**	637	634	512	377	577	504	494
**7. After excluding if did not have valid receipt of help (for mobility/self-care) measured (FINAL SAMPLE)**	637	590	471	368	567	485	473

© 2021 Bressman E et al. *JAMA Network Open.*

eTable 2. Summary of Posthospitalization Care in Study Cohort, Including All Sample Persons Discharged Home From an Acute Care Hospital (Unweighted Percentages)

	**Overall Cohort**	**Cohort Receiving Help**	**Cohort Not Receiving Help**
# of observations	**3591**	**1710**	**1881**
population size			
Received any help within 90 days after discharge, %	1710 (47.6)	1710 (100.0)	0 (0.0)
Received home health within 90 days after discharge, %	1116 (31.1)	744 (43.5)	372 (19.8)
Received help but did not have home health within 90 days after discharge, %	966 (26.9)	966 (56.5)	0 (0.0)

© 2021 Bressman E et al. *JAMA Network Open.*

**eTable 3.** Characteristics of Study Cohort and Care Received After Hospital Discharge, Including All Patients Discharged Home From an Acute Care Hospital, by Year (Weighted Percentages)								
		**2011**	**2012**	**2013**	**2014**	**2015**	**2016**	**2017**
**# of observations**		637	590	471	368	567	485	473
**Estimated population size**		2,513,658	2,755,250	2,597,937	2,385,137	2,479,771	2,455,110	2,716,732
**Post-hospitalization care**								
**Received any help within 3 months after discharge, n (%)**		272 (38.1)	266 (39.2)	218 (38.3)	191 (47.0)	267 (45.8)	251 (49.0)	245 (51.5)
**Had home health visits within 3 months after discharge, n (%)**		171 (24.5)	178 (26.4)	144 (27.4)	102 (26.0)	188 (28.9)	168 (31.7)	165 (33.0)
**Received help but did not have home health visits within 3 months after discharge, n (%)**		154 (22.1)	152 (22.5)	126 (22.6)	118 (29.6)	144 (26.2)	140 (28.4)	132 (28.1)
**Patient characteristics**								
**Female, n (%)**		361 (54.3)	338 (53.8)	258 (50.9)	182 (46.5)	320 (54.3)	280 (55.8)	276 (57.2)
**Age, mean (SD)**		78.0 (6.3)	79.2 (7.0)	79.1 (7.2)	79.0 (6.9)	78.1 (6.7)	78.3 (7.1)	77.9 (7.3)
**Race and ethnicity, n (%)**	White, non-Hispanic	418 (80.0)	408 (81.2)	325 (82.2)	254 (82.7)	395 (80.3)	334 (79.2)	341 (83.6)
	Black, non-Hispanic	158 (9.4)	133 (9.1)	107 (8.9)	87 (9.1)	117 (7.1)	98 (7.2)	97 (7.2)
	Hispanic	31 (5.2)	32 (5.7)	23 (4.6)	**	26 (6.1)	19 (5.7)	16 (4.4)
**Married/living with partner, n (%)**		299 (55.6)	249 (50.4)	213 (54.3)	174 (57.9)	268 (54.9)	224 (55.5)	220 (55.4)
**Self-rated health, n (%)**	Excellent	*	36 (6.9)	32 (8.3)	20 (6.2)	19 (6.6)	28 (5.8)	23 (6.1)
	Very Good	*	102 (18.6)	81 (20.7)	80 (25.6)	66 (26.7)	95 (23.5)	101 (23.7)
	Good	*	179 (31.3)	153 (34.0)	118 (31.6)	101 (31.1)	177 (37.6)	183 (38.7)
	Fair	*	175 (27.9)	133 (24.8)	107 (28.4)	87 (25.5)	127 (22.3)	118 (21.9)

© 2021 Bressman E et al. *JAMA Network Open.*

	Poor	*	86 (14.1)	56 (11.4)	33 (7.8)	33 (10.0)	56 (10.9)	48 (9.6)
**Independent in…, n (%)**	Mobility	*	403 (70.7)	328 (75.3)	269 (77.8)	212 (71.8)	331 (71.4)	334 (71.7)
	Self-care	*	460 (82.2)	378 (84.8)	302 (84.2)	242 (81.5)	381 (81.2)	380 (81.6)
	Household activities	*	333 (61.8)	263 (64.4)	222 (67.1)	176 (61.9)	268 (60.8)	269 (60.8)
	Medical care	*	461 (82.2)	353 (79.6)	280 (80.9)	248 (85.0)	379 (81.7)	380 (83.8)
**Cognitive status, n (%)**	No or Possible Impairment	*	470 (84.6)	306 (84.3)	291 (84.6)	259 (89.0)	408 (89.3)	391 (88.0)
	Probable Dementia	*	108 (14.0)	96 (14.2)	68 (14.0)	47 (11.0)	75 (10.5)	82 (12.0)
**Hospital length of stay in days, mean (SD)**		3.7 (3.4)	3.4 (3.0)	3.5 (3.0)	3.6 (2.5)	3.6 (3.3)	3.4 (2.7)	3.4 (2.5)
**Diagnosis Related Group, n (%)****	Major joint replacement (470)	22 (4.4)	14 (2.9)	15 (4.8)	17 (6.0)	36 (8.8)	30 (8.5)	27 (10.6)
	Septicemia or severe sepsis (871)	16 (2.3)	**	13 (2.7)	**	19 (3.2)	19 (4.0)	17 (2.4)
	Esophagitis, gastroenteritis and miscellaneous digestive disorders (392)	16 (2.3)	15 (2.3)	12 (2.4)	**	15 (2.3)	13 (2.4)	13 (3.0)
	Simple pneumonia & pleurisy (194)	**	13 (1.8)	**	15 (3.8)	13 (2.7)	**	**
	Cellulitis (603)	12 (2.3)	**	**	**	**	12 (2.8)	**

* Characteristic measured in the survey prior to hospitalization and is thus missing in the first year of the study period.
** Cells with fewer than 11 respondents

© 2021 Bressman E et al. *JAMA Network Open.*

**eTable 4.** Characteristics of Study Cohort, Including All Patients Discharged Home From an Acute Care Hospital (Unweighted Percentages)				
	**Level**	**Overall Cohort**	**Cohort Receiving Help**	**Cohort Not Receiving Help**
**# of observations**		**3591**	**1710**	**1881**
**Female, n (%)**		2015 (56.1)	1092 (63.9)	923 (49.1)
**Age, mean (SD)**		81.2 (7.5)	82.7 (7.8)	79.7 (6.8)
**Race and ethnicity, n (%)**	White, non-Hispanic	2475 (68.9)	1114 (65.1)	1361 (72.4)
	Black, non-Hispanic	797 (22.2)	409 (23.9)	388 (20.6)
	Hispanic	165 (4.6)	97 (5.7)	68 (3.6)
	Other	154 (4.3)	90 (5.3)	64 (3.4)
**Married/living with partner, n (%)**		1647 (46.3)	722 (42.7)	925 (49.5)
**Self-rated health, n (%)**	Excellent	158 (5.9)	55 (4.2)	103 (7.5)
	Very Good	525 (19.6)	188 (14.4)	337 (24.7)
	Good	911 (34.0)	411 (31.4)	500 (36.6)
	Fair	747 (27.9)	425 (32.4)	322 (23.6)
	Poor	312 (11.7)	216 (16.5)	96 (7.0)
**Independent in…, n (%)**	Mobility	1877 (69.7)	738 (56.0)	1139 (82.8)
	Self-care	2143 (79.6)	888 (67.4)	1255 (91.2)
	Household activities	1531 (56.9)	485 (36.8)	1046 (76.0)
	Medical care	2101 (78.0)	850 (64.5)	1251 (90.9)
**Cognitive status, n (%)**	No or Possible Impairment	2179 (80.9)	923 (70.0)	1256 (91.3)
	Probable Dementia	476 (17.7)	371 (28.2)	105 (7.6)
**Hospital length of stay in days, mean (SD)**		3.7 (3.2)	4.0 (3.5)	3.3 (2.9)
**Diagnosis Related Group, n (%)**	Major joint replacement (470)	162 (4.5)	88 (5.1)	74 (3.9)
	Septicemia or severe sepsis (871)	102 (2.8)	68 (4.0)	34 (1.8)

© 2021 Bressman E et al. *JAMA Network Open.*

	Esophagitis, gastroenteritis and miscellaneous digestive disorders (392)	93 (2.6)	54 (3.2)	39 (2.1)
	Simple pneumonia & pleurisy (194)	75 (2.1)	29 (1.7)	46 (2.4)
	Cellulitis (603)	69 (1.9)	22 (1.3)	47 (2.5)

© 2021 Bressman E et al. *JAMA Network Open.*

eFigure 1. Percentage of Sample Persons Discharged From an Acute Care Hospitalization Who Received ADL Help After Discharge and Persons Who Received ADL and No Home Health Care After Discharge, by Year (Unweighted)

A: Persons Receiving ADL Help After Discharge

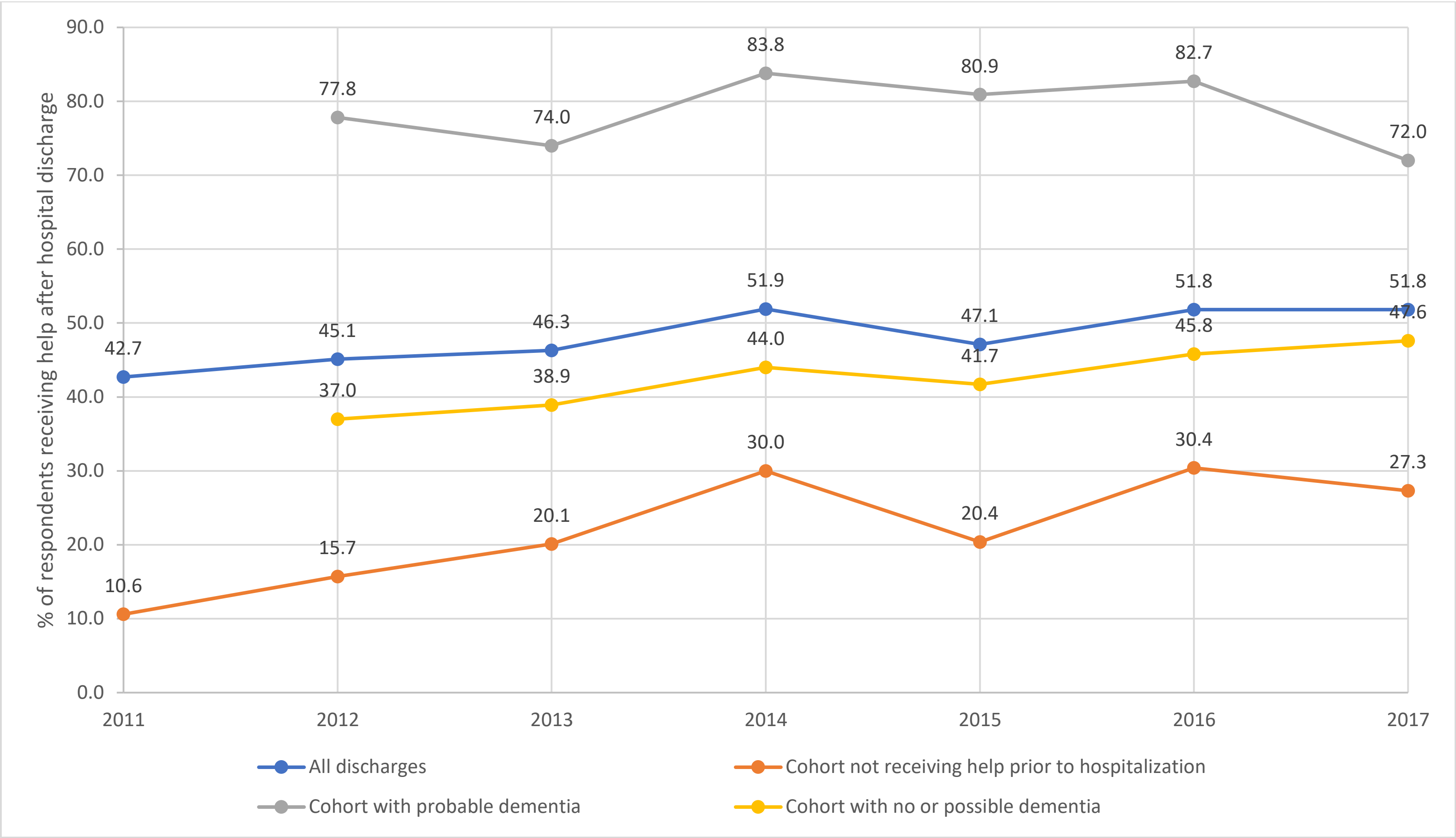

© 2021 Bressman E et al. *JAMA Network Open.*

Appendix Figure 1b: Persons Receiving ADL Help and No Home Health After Discharge

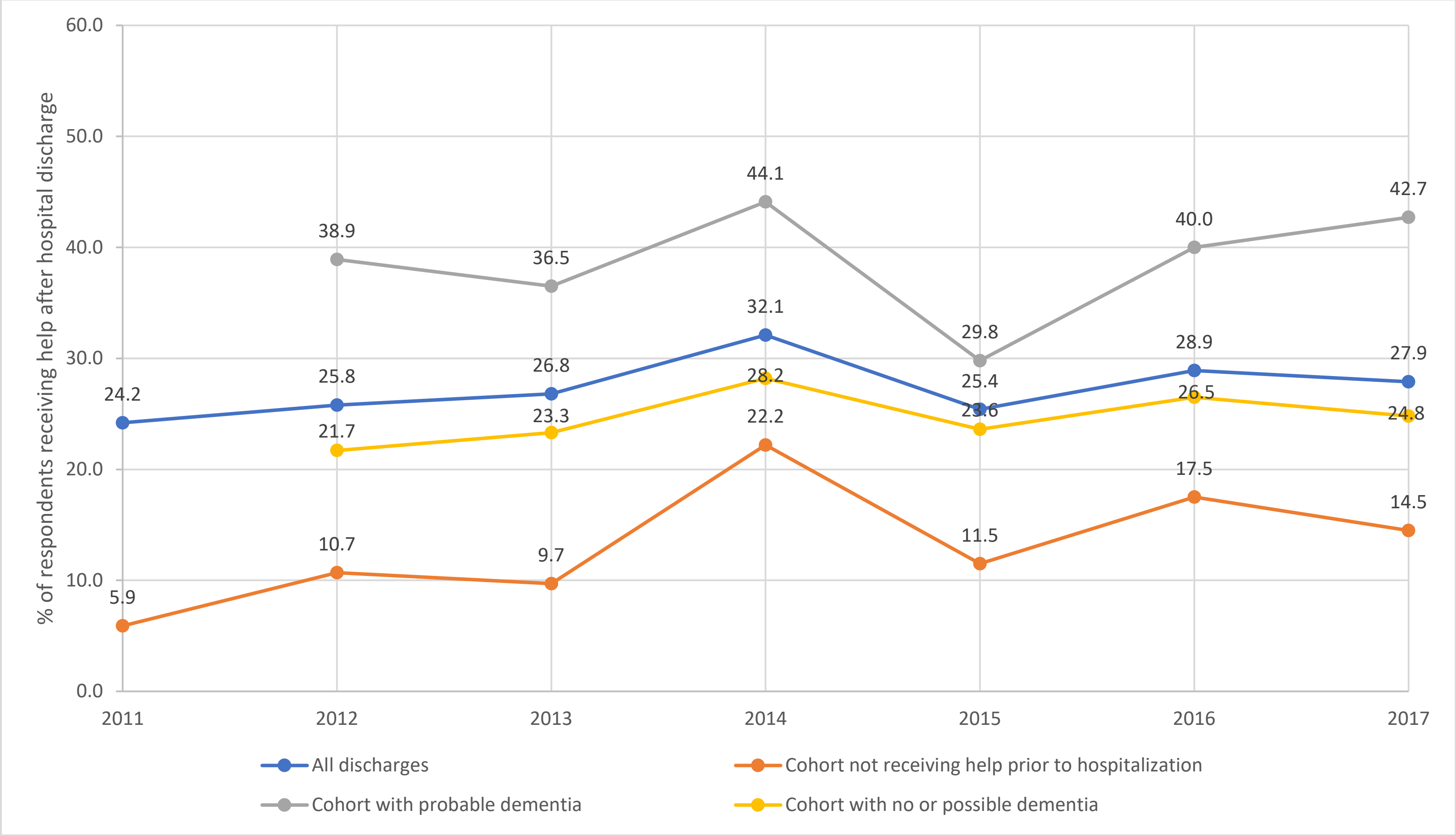

© 2021 Bressman E et al. *JAMA Network Open.*

eFigure 2. Weighted Percentage of Sample Persons Discharged From an Acute Care Hospitalization Who Received ADL Help After Discharge, by Year and by Survey Wave

A. Persons Discharged From an Acute Care Hospitalization Who Received ADL Help After Discharge

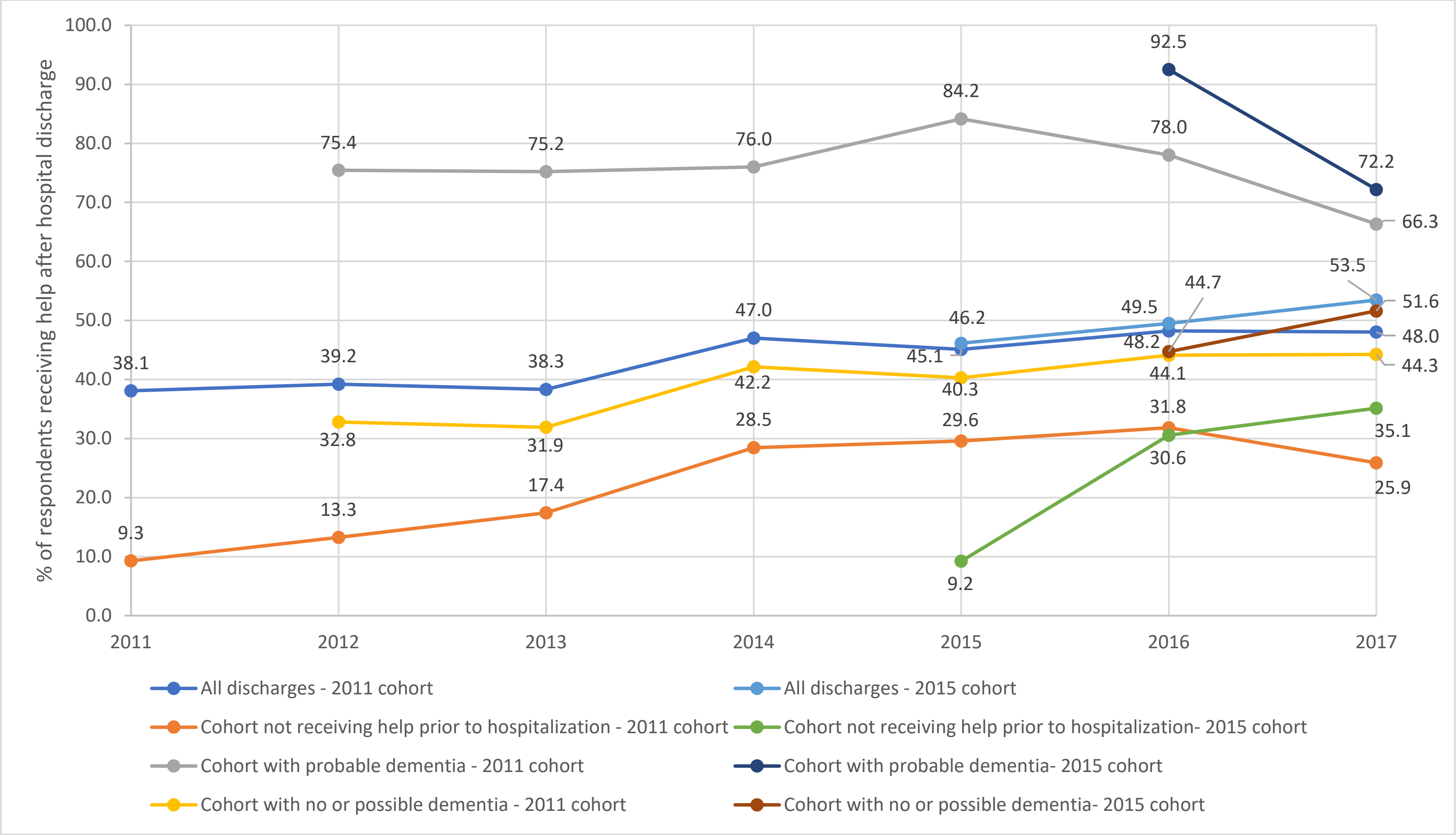

© 2021 Bressman E et al. *JAMA Network Open.*

B: Persons Discharged From an Acute Care Hospitalization Who Received ADL Help and No Home Health After Discharge

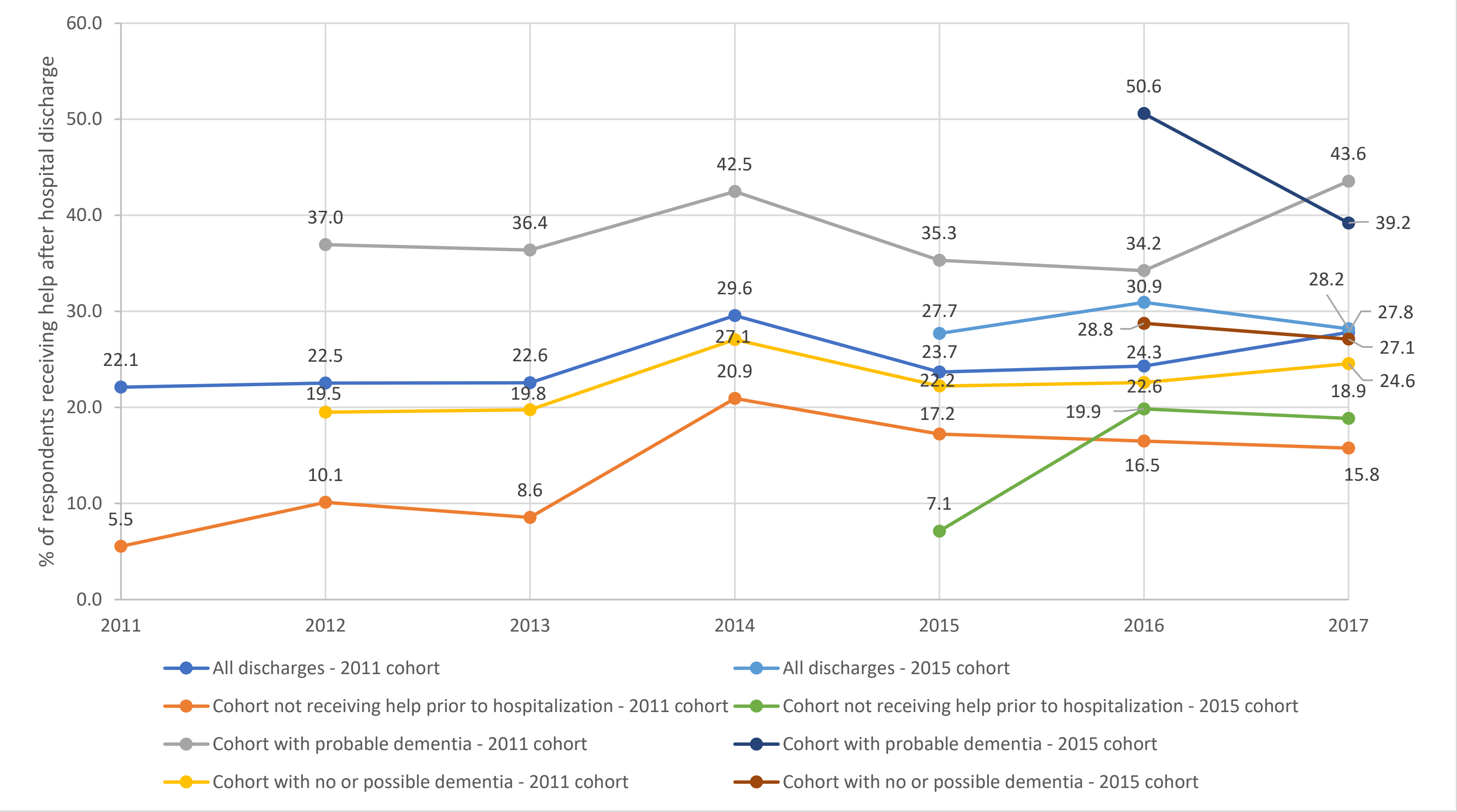

© 2021 Bressman E et al. *JAMA Network Open.*

eFigure 3. Unweighted Percentage of Individuals Who Reported Receiving Help in Each Month After Hospital Discharge and Those Who Received Help and Did Not Have Any Medicare-Reimbursed Home Health Visits in Those 3 Months

A. Individuals Who Reported Receiving Help in Each Month After Hospital Discharge

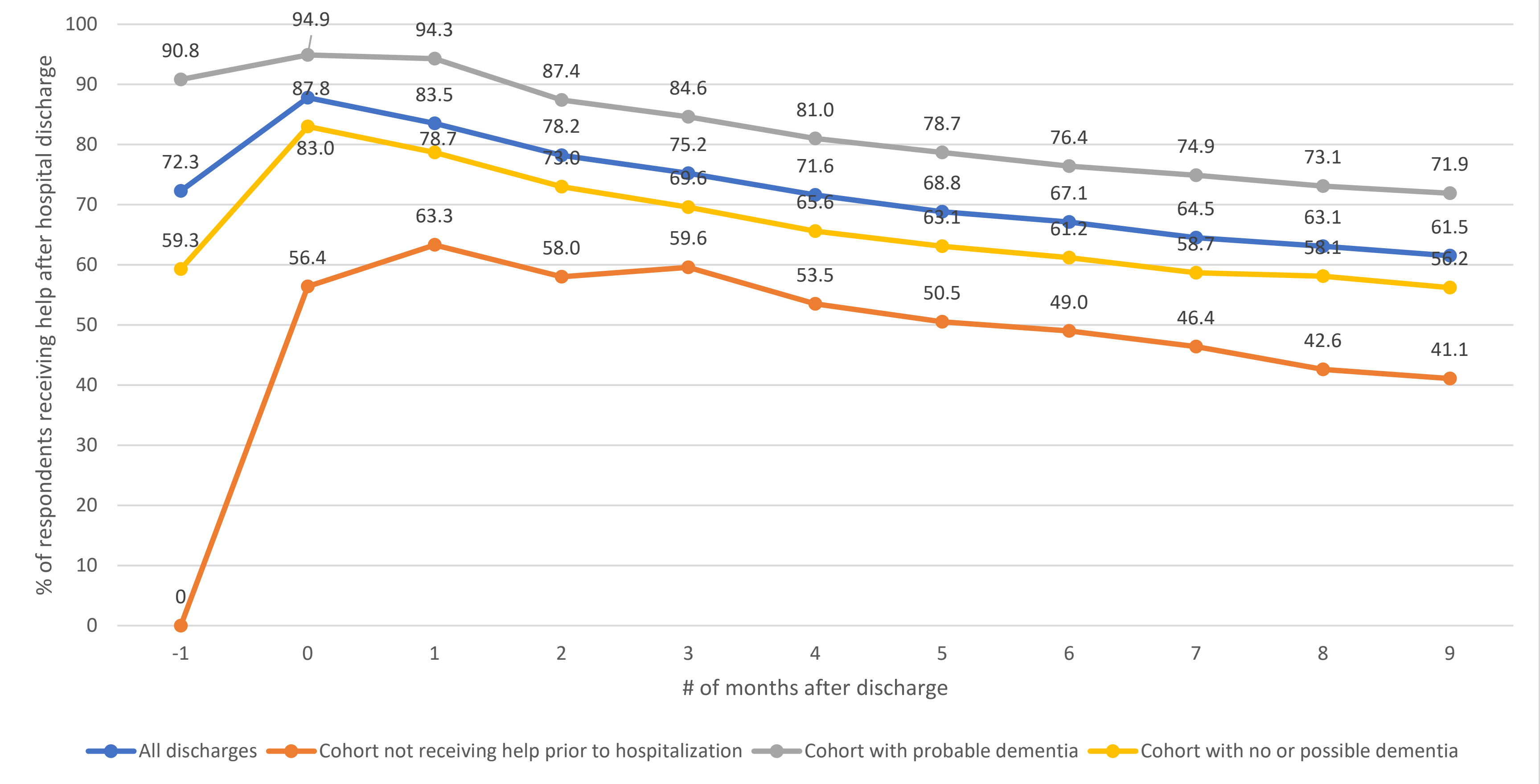

© 2021 Bressman E et al. *JAMA Network Open.*

B: Individuals Who Received Help and Did Not Have Any Medicare-Reimbursed Home Health Visits in Those 3 Months

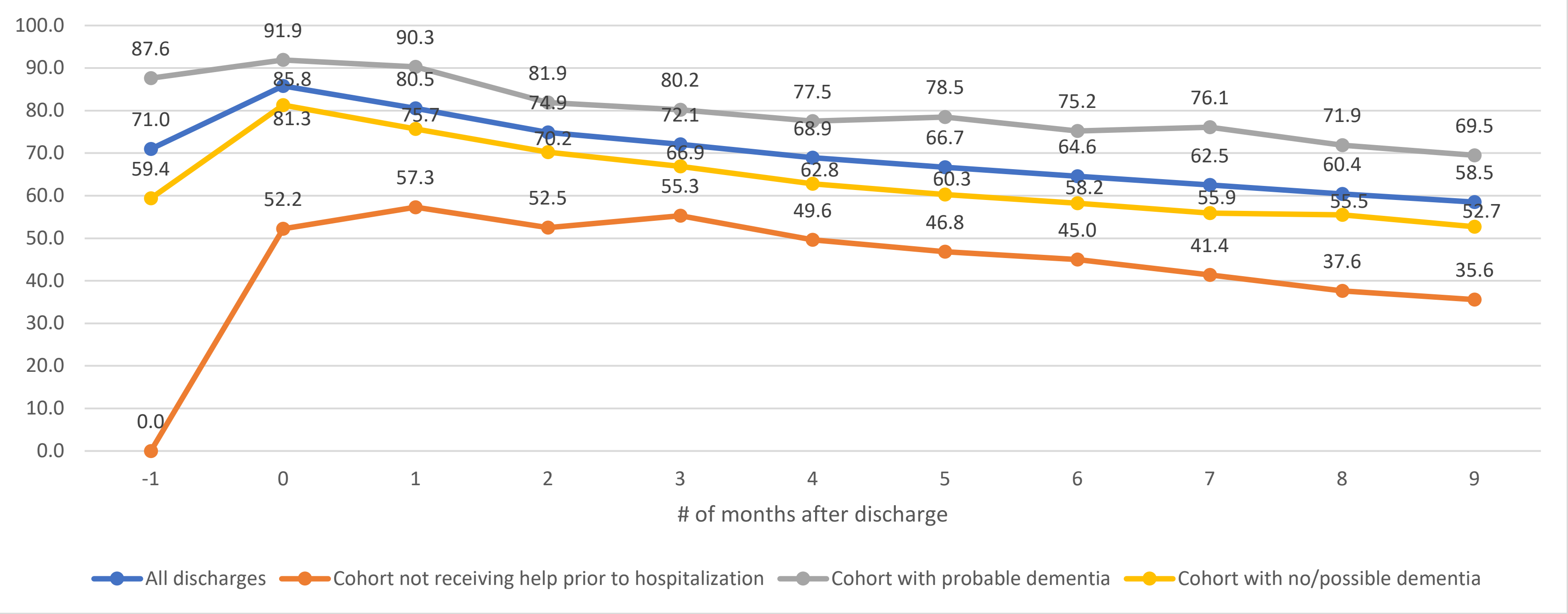

© 2021 Bressman E et al. *JAMA Network Open.*
